# Supplementary material for: Deciphering Promoter Hypermethylation of Genes Encoding for RASSF/Hippo Pathway Reveals the Poor Prognostic Factor of RASSF2 Gene Silencing in Colon Cancers
Source: Cancers (Basel). 2021 Nov 26;13(23):5957. doi: 10.3390/cancers13235957 (PMC8656858; doi:10.3390/cancers13235957)
Supplement: Supplementary file 1 [file cancers-13-05957-s001.zip › cancers-1447915-supplementary.pdf]

Supplementary Material

Deciphering Promoter Hypermethylation of Genes Encoding for RASSF/Hippo Pathway Reveals the Poor Prognostic Factor of RASSF2 Gene Silencing in Colon Cancers

Marc Riffet, Yassine Eid, Maxime Faisant, Audrey Fohlen, Benjamin Menahem, Arnaud Alves, Fatéméh Dubois, Guénaelle Levallet and Céline Bazille

Table S1. List of primers used for MS-PCR for methylated (M) and non-methylated (U) genes.

RASSF1A	**Primer sequences**	**Tm(°C)**
U	F: 5'-TTTGGTTGGAGTGTGTTAATGTG-3'<br>R: 5'-CAAACCCCACAAACTAAAAACAA-3'	60
M	F: 5'-GTGTTAACGCGTTGCGTATC-3'<br>R: 5'-AACCCCGCGAACTAAAAACGA-3'	62
RASSF2		
U	F: 5'-AGTTTGTTGTTGTTTTTTAGGTGG-3'<br>R: 5'- AAAAAACCAACAACCCCCACA-3'	63
M	F: 5'-GTTCGTCGTCGTTTTTTAGGCG-3'<br>R: 5'- AAAAACCAACGACCCCCGCG-3'	63
STK4		
U	F: 5'-TTTGTGGGGTGGGTTTAGGAGGTTTGT-3'<br>R: 5'-AACCAATAACCCCTCACCAACACAACAA-3'	63
M	F: 5'-GCGGGGCGGGTTTAGGAGGTTC-3'<br>R: 5'-CCAATAACCCCTCACCGACGC-3'	63
STK3		
U	F: 5'-TTTTAAGTGGGAGGGAGATTTGTTGTGG-3'<br>R: 5'-AAAAACCAAAACACCAACCAACCAAACC-3'	61
M	F: 5'-CGGGAGGGAGATTCGTCGCG-3'<br>R: 5'-AAACCGAAACACCGACCGACCG-3'	63
LATS1		
U	F: 5'-TGAATGATTAGAGTTGTGGGTGATGT-3'<br>R: 5′-AAACATTTCCCAACATCACTTACACA-3′	60
M	F: 5′-GAACGATTAGAGTTGCGGGCGAC-3′<br>R: 5′-AACATTTCCCGACGTCGCTTACG-3′	62
LATS2		
U	F: 5′-GGTGTTTTGTTTGGATTGGTATGTGGTT-3′<br>R: 5′-CATCTTCCCAAAACACTCACACCACA-3′	60
M	F: 5′-TTCGTTCGGATTGGTATGCGGTC-3′<br>R: 5′-CCATCTTCCCGAAACGCTCACG-3′	60

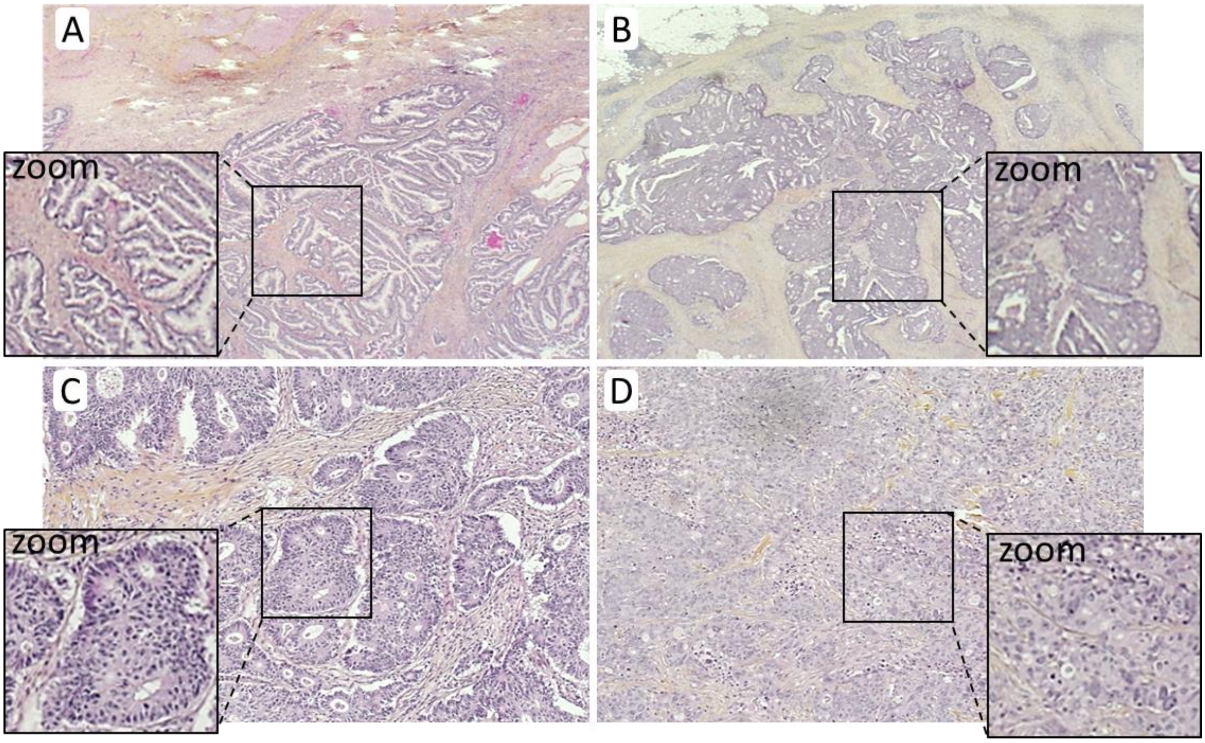

Figure S1. Illustrations of the different grades of differentiation of adenocarcinomas. (**A**) well differentiated adenocarcinoma: well individualized glandular structures, more or less branched (HES ×20). (**B** and **C**) Moderately differentiated adenocarcinoma: glandular structures of cribriform appearance, appearance of fused glands (HES ×20 and ×100). (**D**) Poorly differentiated adenocarcinoma: formation of clumps, with rare persistent glandular lumens (HES ×100).

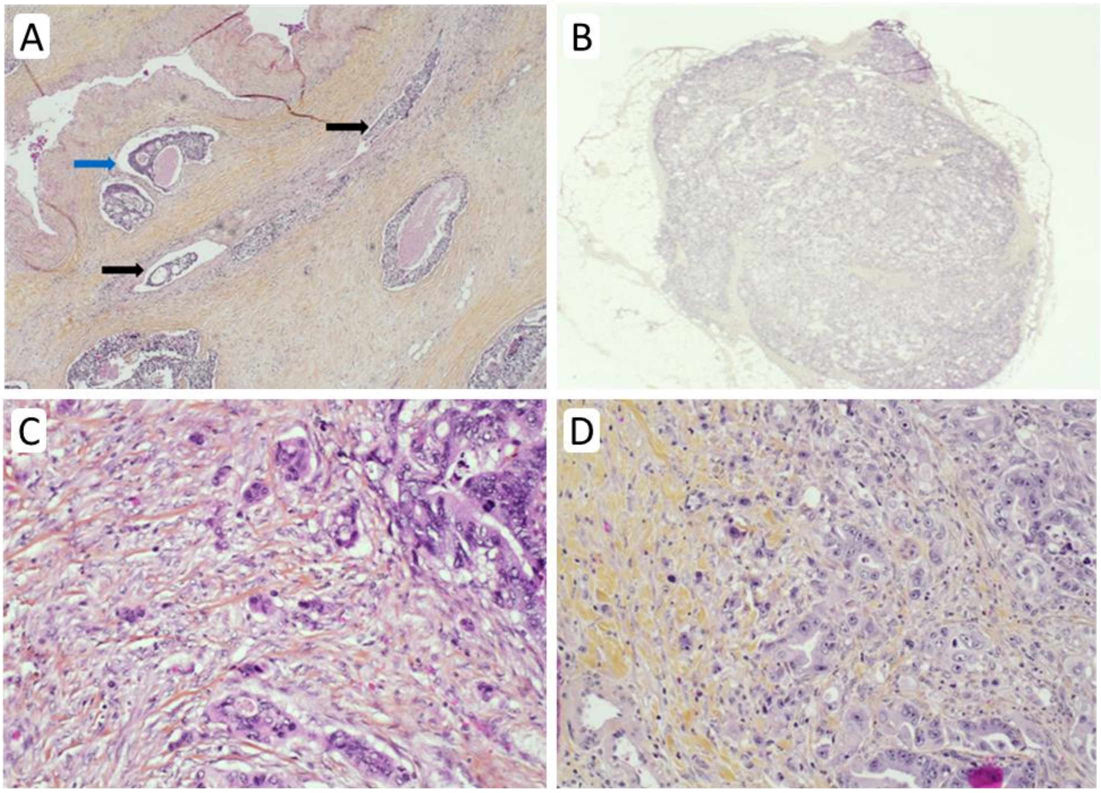

Figure S2. Illustration of the histological criteria of the cohort. (**A**) Tumor venous and lymphatic emboli. Tumor cell clusters are located in lymphatics (blue arrow), as well as in venous (black arrows) (HES ×50). (**B**) Illustration of a tumor deposit located in the mesocolon without identifiable lymph node parenchyma (HES ×20). (**C**) Illustration of grade 2 budding: a few isolated cells or clusters of less than 5 cells are seen detaching from the tumor glands (HES ×200). (**D**) Grade 3 budding: many single cells or clusters of less than 5 cells are detached from the tumor glands (HES ×200).

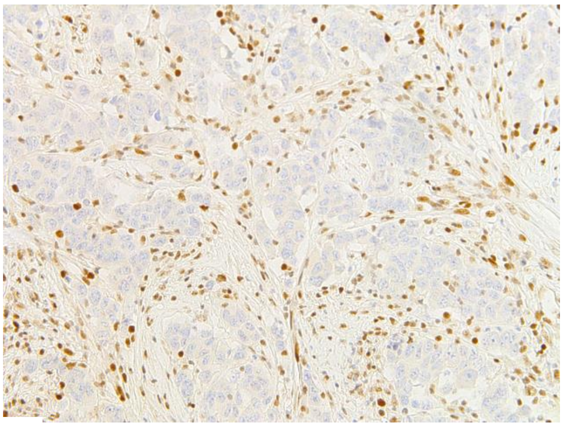

A

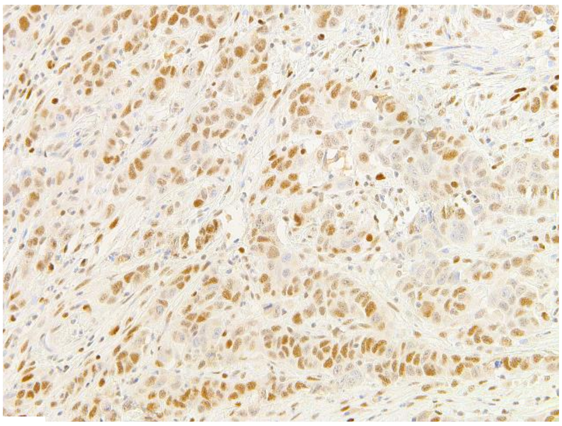

B

Figure S3. Illustration of deficient MMR (dMMR) status. (**A**) Example of a loss of MLH1 expression in favor of a dMMR-IHC (×20). (**B**) The same specimen with expression of MSH2 (×20).

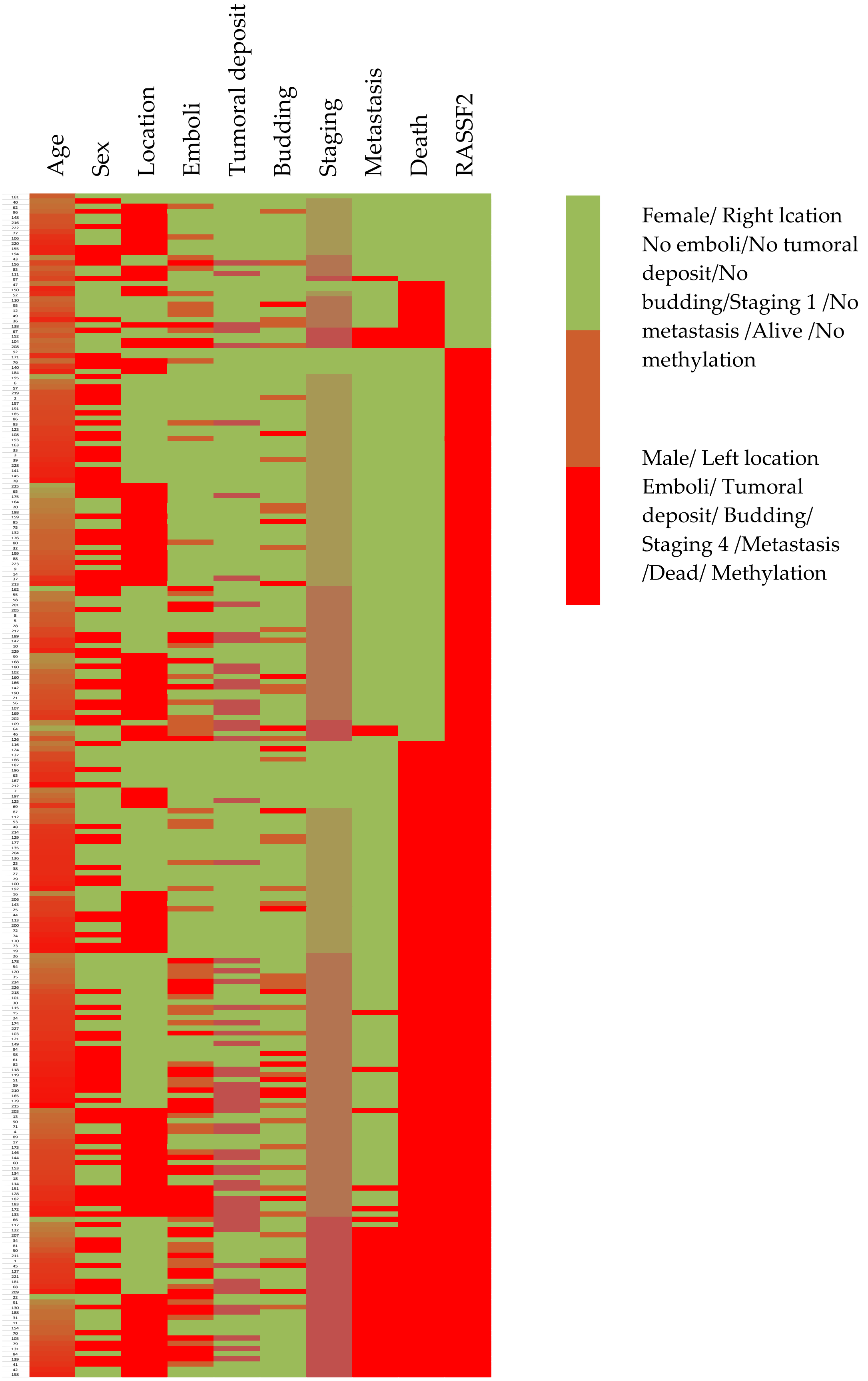

Figure S4.: Heat map of clinical and histological data of the cohort.

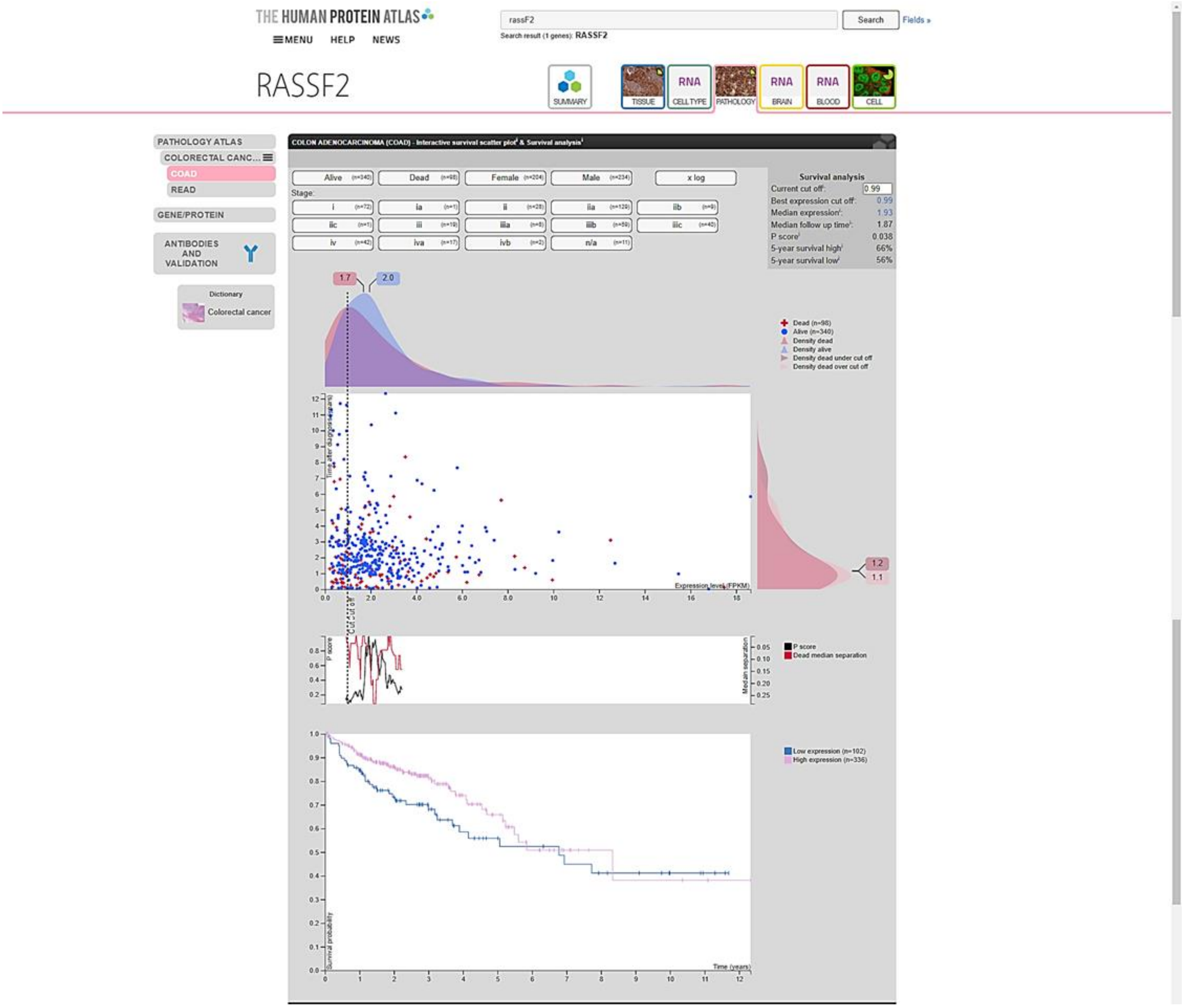

Figure S5: Low RASSF2 mRNA expression predicts significant worse overall survival in 438 patients with colon cancer from the Table 336. or weakly (n = 102) expresses the RASSF2 messenger. Survival analysis reveals that the low RASSF2 mRNA expression predicts significant worse overall survival in 438 patients with colon cancer (P score: 0.038, Expression of RASSF2 in colorectal cancer—The Human Protein Atlas).
